# Expanding black soldier fly (BSF; *Hermetia illucens*; Diptera: Stratiomyidae) in the developing world: Use of BSF larvae as a biological tool to recycle various organic biowastes for alternative protein production in Nepal

**DOI:** 10.1016/j.btre.2025.e00879

**Published:** 2025-02-10

**Authors:** Bhola Gautam, Sundar Tiwari, Min Raj Pokhrel, Jeffery K. Tomberlin, Prabhat Khanal

**Affiliations:** a Department of Entomology, Faculty of Agriculture, Agriculture and Forestry University, Nepal; b Animal Science, Production and Welfare Division, Faculty of Biosciences and Aquaculture, Nord University, Norway; c Department of Entomology, Texas A&M University, USA

**Keywords:** Alternative feed, Black soldier fly, Growth performance, Nutrient composition, Rapeseed cake supplementation, Waste management

## Abstract

The growing global demand for food, particularly animal protein, is intensifying challenges related to food security and environmental sustainability. The increase in organic waste generation, coupled with inefficient waste management, is further deteriorating living conditions by negatively impacting the environment and public health, especially in developing nations. This study investigated the potential of black soldier fly larvae (BSFL) to recycle major daily organic waste fractions in Nepal. BSFL were exposed to seven different biowaste-based substrates locally sourced from fruit and vegetable markets, farms, and food industries. Additionally, the study evaluated rapeseed cake as a supplement to enhance BSFL growth and nutritional content. BSFL survival rates exceeded 80 % on food industry waste but dropped to 63 % on high-moisture substrates like vegetable waste. Mixed vegetable waste (14.7 mg/day) and bakery waste (11.5 mg/day) supported higher average daily weight gain, likely due to their better nutrient values (soluble carbohydrates and proteins). Although rapeseed cake alone hindered larval growth, its supplementation to biowastes improved growth, survival, and bioconversion rates, increased larval protein content up to 32 %, and reduced fat by 36 %. These findings indicate BSFL can effectively recycle diverse, locally available organic wastes in developing countries like Nepal, providing a sustainable source of domestic protein and contributing to feed security. As this is the first BSFL study in Nepal, further research is needed to elucidate the chemical and microbial safety of BSFL reared on biowastes and to develop technical solutions for commercial BSFL production in countries with a low-income economy.

## Highlights

• This is the first study to evaluate the performance and composition of BSF larvae on local organic wastes and byproducts in Nepal.
• Most organic wastes locally available in Nepal were suitable for BSF larvae production.
• The physical and chemical composition of feeding substrates influenced BSF larval performance.
• Supplementing biowastes with rapeseed cake improved the growth and protein content of BSF larvae, however rapeseed cake alone did not support larval growth.

## Graphical abstract

**Figure:**
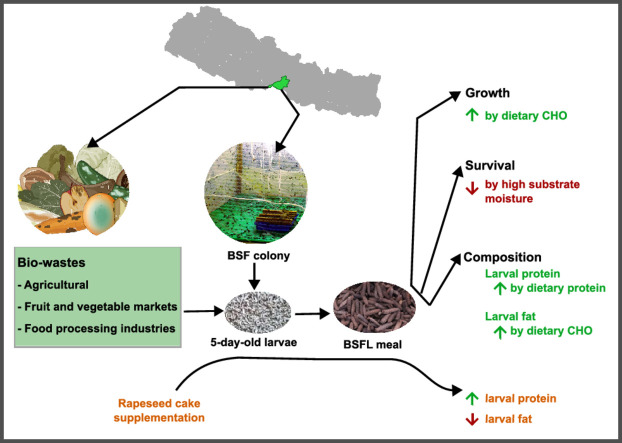

## 1 Introduction

Animal feed production in many countries of the Global South often relies on human-edible raw materials, increasing competition for human food and animal feed resources [68]. Improving self-sufficiency in domestic feed production is a common challenge. In Nepal, there is a poor domestic supply of raw ingredients for feed production, and feed industries heavily rely on imports to meet their demand [26]. This situation highlights the urgent need to find alternative feeding resources that can be locally produced sustainably. In recent decades, insects have been recognized as a potential alternative feed ingredient due to their high nutritional quality, good bio-conversion efficiency of low-grade organic resources, and low environmental impacts [2,42]. Among the insects used for such purposes, the black soldier fly (BSF; *Hermetia illucens* (L.) Diptera: Stratiomyidae) has gained interest, and is commercially produced globally [55]. Black soldier fly larvae (BSFL) are rich in protein and fat, can grow on diverse organic wastes, and efficiently convert those wastes into high-quality biomass for use as a livestock and aquaculture feed ingredient [61,65]. However, to date, no studies have evaluated the potential exploration of BSFL as a biological tool for recycling local biowastes and by-products in Nepal, despite the significant environmental and public health concerns arising from poor handling and management of municipal and other organic solid waste generated [54].

Growth, survival, conversion efficiency, and the nutritional composition of BSFL are influenced by the type of organic substrates used to rear them [25,40,59]. Different characteristics of the feeding substrate, including texture, nutrient content, and moisture level, can significantly influence larval development and nutrient utilization [44,53,59,69]. Multiple studies indicate that BSFL can efficiently recycle common municipal biowastes [20,28,36,43,45]. Nepal generates 419 metric tons of municipal solid waste daily, with approximately 0.18 kg of waste generated per capita, and around 70 % of the waste being organic [1,51]. Significant portions of organic waste are also generated on farm, though these remain undocumented. Poor waste management, including illegal dumping and inefficient disposal methods like landfilling, has raised environmental pollution and public health risks in urban areas [54]. These biowastes can vary in chemical and nutritional composition depending on their type and source; thus, evaluating their suitability for BSFL production is essential. This study hypothesized that most locally available biowastes in Nepal would be suitable for recycling by BSFL, though the growth and nutritional quality of BSFL could vary depending on the type and characteristics of the biowaste.

Understanding the nutritional requirement of BSFL is also crucial for developing a sustainable mass-rearing system, ensuring optimal growth, bioconversion, and larval composition [45]. Although most organic wastes may supply essential nutrients like protein, fat, and carbohydrates to BSFL [31,36], supplementing these organic wastes with nutrient-rich, inexpensive, organic byproducts can maximize the nutritional values of BSFL [56]. Rapeseed cake, a byproduct of the rapeseed (*Brassica campestris* L.) oil refinery industries, is rich in protein content [6] and is available year-round in Nepal. Despite its nutritional value, rapeseed cake is not widely utilized in animal feed due to its poor digestibility [30] and the presence of certain antinutritional compounds such as glucosinolates, phytic acid, and phenolic compounds [29,46]. Nevertheless, such residuals have traditionally been used as a soil amendment in parts of Asia, including China, the second-largest producer of rapeseed [13]. Recent studies have demonstrated the feasibility of BSFL growth when fed rapeseed cake, and its potential to improve the quality of BSFL meals generated. Hoc et al. [32] successfully reared BSFL on chicken feed supplemented with varying levels of rapeseed cake, while Eggink et al. [25] used rapeseed cake as the sole rearing substrate. However, to our knowledge, the use of rapeseed cake to supplement organic wastes for BSFL rearing has yet to be evaluated. In this context, this study further hypothesized that the growth and nutritional composition of BSFL could be improved by supplementing rapeseed cake with regular organic biowastes generated in developing countries like Nepal.

## 2 Materials and methods

All experimental activities in this study were carried out at the laboratory of Department of Entomology, Agriculture and Forestry University (AFU), Chitwan, Nepal, under the collaborative CEER (Circular Economy for Education and Research) project between Nepal and Norway [35] using standard animal experimental guidelines and regulations.

### 2.1 Establishment of BSF colony

**Fig. 1:**
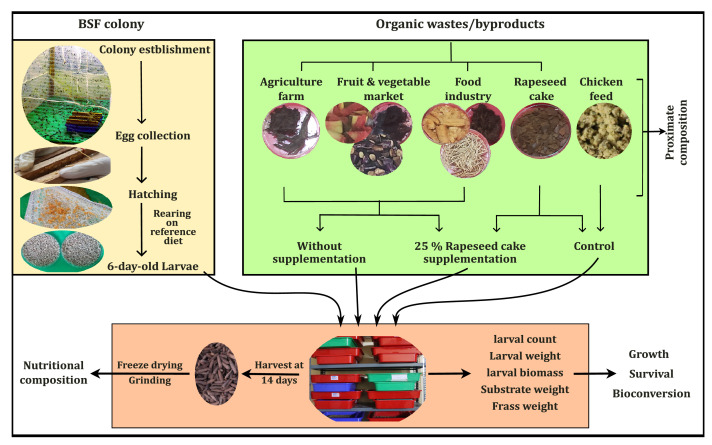
Experimental outline of the study. First, adult and larval colonies of black soldier fly (BSF) were established to obtain 6-day-old larvae. These larvae were then exposed to biowastes from agricultural farms, fruit and vegetable markets, and food industries with or without 25 % rapeseed cake supplementation for 14 days. In addition, rapeseed cake and broiler chicken feed were used as controls. During the feeding trial, data on initial and final larval and substrate weight and larval count were recorded to evaluate growth, survival, and bioconversion. After 14 days of biowaste treatment, 20-day-old larvae were harvested and analyzed for various nutritional compositions. For details on experimental treatments, see Table 1.

The BSF colonies (larvae and adult) were maintained at 50 to 70 % relative humidity and 28 ± 2 °C temperature at the laboratory of Department of Entomology, AFU, Chitwan, Bagmati Province, Nepal (Fig. 1). To initiate the colony, fourth instar BSFL were obtained from a local agriculture farm. These larvae were reared in plastic containers (20 × 30 × 16 cm^3^) with chicken feed for two generations to establish a stable BSF colony of ∼5000 adult flies. First, the prepupae developed in these containers were collected and transferred to dark cages (1 × 1 × 1 m^3^) for pupation. Emerging adults were then transferred to rearing cages (1.5 × 1.5 × 1.5 m^3^) made of nylon net (12 mesh size), with ample natural daylight through windows. Inside each cage, a plastic container (20 × 30 × 16 cm^3^) half-filled with fermented chicken feed and covered by steel wire mesh (size: 10) was placed to attract female BSF adults for egg-laying. On the top of this steel mesh, wooden pieces made of Sal (*Shorea robusta* Gaertn. f.) wood (20 × 5 × 1 cm^3^) were tied and stacked together, leaving a ∼2 mm slit between them to serve as egg-laying sites. The eggs were then collected every two days and transferred to new containers (20 × 30 × 16 cm^3^) supplied with chicken feed, where they were allowed to hatch into larvae. These containers were changed daily and labeled with the hatching date to ensure all larvae were of similar ages. BSF eggs obtained from this colony were hatched, and the larvae were reared on chicken feed for five days before exposing them to various experimental substrates as described below.

### 2.2 Selection of feeding substrates (biowaste/byproducts) for BSFL

Seven types of locally available organic waste from agriculture farms, fruit and vegetable markets, and food processing industries were used as the feeding substrates for BSFL (Table 1): banana pseudo-stem waste (BPW), mixed fruit waste (MFW), mixed vegetable waste (MVW), cowpea vegetable waste (CVW), bakery food waste (BFW), chowmein food waste (CFW), and rumen digesta waste (RDW). These organic wastes were used as sole-feeding substrates (BPW, MFW, MVW, CVW, BFW, CFW, and RDW) or with 25 % rapeseed cake (RSC) supplementation (BPW_RSC, MFW_RSC, MVW_RSC, CVW_RSC, BFW_RSC, CFW_RSC, and RDW_RSC). Additionally, a broiler chicken feed (BCF) and a 100 % rapeseed cake (RSC) were used as positive controls to compare the growth performance and nutritional composition of BSFL exposed to biowastes with or without rapeseed cake supplementation, totaling 16 different feeding treatments (Table 1). Broiler chicken feed was selected due to its widespread use in BSFL growth trials and its consistency in producing comparable results across studies [10,14,21,47]. A representative pooled sample of each substrate was taken and stored at -80 °C for subsequent proximate analyses.

**Table 1:** An overview of the composition of organic waste used as feeding substrates/treatments for black soldier fly (BSF) larvae rearing.

**SN**	**Feeding substrates**	**Abbreviation**	**Type**	**Composition**	**Source**	**Preparation methods**
1	Banana pseudo-stem waste	BPW	Agricultural waste	Pseudo-stem of the banana plant after harvesting Fruit	Horticulture farm, AFU, Bharatpur	Finely chopped, homogenized using a feed crusher and mixer hammer mill (Kingrunda-9FC-360, China), and left in a shaded room for 2–3 days at a temperature of 28 ± 5 °C and used as available
2	Mixed fruit waste	MFW	Fruit and vegetable wastes	Watermelon and banana (1:1)	Bharatpur Metropolitan City Fruit and Vegetable Market	
3	Mixed vegetable waste	MVW		Potato, tomato and brinjal (2:1:1)		
4	Cowpea vegetable waste			Green cowpea pods		
5	Bakery food waste	BFW	Food industry wastes (food processing)	Expired bread and bread cuttings leftovers from slicing	Local bakery	Soaked in water 1 day before treatment to maintain approximately 70 % moisture
6	Chowmein food waste	CFW		Date expired Chowmein	Local chowmein Industry, Bharatpur	
7	Rumen digesta waste	RDW		Discarded rumen content of buffalo after slaughtering	Local slaughterhouse, Bharatpur	Used as available. and left in a shaded room for 2–3 days at a temperature of 28±5 °C to dry out excess moisture
8	Banana pseudo-stem waste + Rapeseed cake	BPW_RSC	Organic wastes supplemented with 25 % rapeseed cake	Banana pseudo-stem waste (BPW) 75 % and Rapeseed cake (RSC) 25 %	The same organic wastes from treatments 1–7 and rapeseed cake from a local oil refinery	Organic wastes and rapeseed cake were mixed in the stated ratio and homogenized by hand 1 day before treatment
9	Mixed fruit waste + Rapeseed cake	MFW_RSC		Mixed fruit waste (MFW) 75 % and Rapeseed cake (RSC) 25 %		
10	Mixed vegetable waste + Rapeseed cake	MVW_RSC		Mixed vegetable waste (MVW) 75 % and Rapeseed cake (RSC) 25 %		
11	Cowpea vegetable waste + Rapeseed cake	CVW_RSC		Cowpea vegetable waste (CVW) 75 % and Rapeseed cake (RSC) 25 %		
12	Bakery food waste + Rapeseed cake	BFW_RSC		Bakery food waste (BFW) 75 % and Rapeseed cake (RSC) 25 %		
13	Chowmein food waste + Rapeseed cake	CFW_RSC		Chowmein food waste (CFW) 75 % and Rapeseed cake (RSC) 25 %		
14	Rumen digesta waste + Rapeseed cake	RDW_RSC		Rumen digesta waste (RDW) 75 % and Rapeseed cake (RSC) 25 %		
15	Rapeseed cake	RSC	Control	Rapeseed cake (100 %)	Local rapeseed oil refinery	Soaked in water 1 day before treatment to maintain approximately 70 % moisture
16	Broiler chicken	BCF	Control	Broiler chicken feed (Broiler starter ration, Global Agro Products, Bharatpur-3-Chitwan)	Local Poultry feed industry, Bharatpur	

### 2.3 Rearing of young BSFL to various feeding substrates

Six-day-old BSFL obtained from the colony development unit were reared in 16 experimental substrates in plastic containers (20 × 30 × 16 cm) (Fig. 1) with three independent replicates (N = 3). Larvae were manually counted on a petri-plate, and 3600 larvae were released in each rearing container to maintain a uniform rearing density of 6 larvae/cm^2^ across treatments. Feeding substrates were allocated based on dry matter (DM) basis to maintain a similar feeding rate of 25 mg DM feed/larva/day across all treatments, as recommended previously [23]. The larvae were fed to experimental substrates for 14 days (until the larval age of 20 days), and the feeding experiment was carried out at 50 % - 70 % relative humidity and 28 ± 2 °C temperature. Experimental substrates were added to larval rearing containers on days 0, 5, 8, and 11, with 25 % of the total substrates added during each feeding. The larvae were harvested at 14 days of experimental feeding and then were cleaned, packed in zip lock bags, and stored at -80 °C until freeze-drying. The larval samples were dried using a freeze-dryer (BIOBASE Tabletop Freeze Dryer, Model: BK-FD12S, China), and afterward, they were ground, vacuum-packed, and subjected to various chemical and nutritional analyses.

### 2.4 The evaluation of the growth and performance of BSFL

In this study, the growth, survival, waste reduction, and bioconversion of BSFL were evaluated under the feeding substrate mentioned above. For this, 60 BSFLs were randomly selected from each container (180 BSFL per treatment) and weighed on each alternate day from 6 to 20 days of larval age using a Phoenix WT3203 scale (Garbsen, Germany; precision: 0.001 g). After weighing, the larvae returned to their respective feeding containers. The larval growth data were analyzed using a logistic growth model to capture the complex non-linear biological growth pattern observed in BSFL [38,63,64].

(1) $$W_{t}=\frac{k}{1+\left(\frac{k-N_{0}}{N_{0}}\right)e^{-r.t}}$$

Where, W_t_ represents the larval weight at time *t, k* is the carrying capacity (maximum weight attainable), N_0_​ is the initial weight, and *r* is the maximal specific growth rate.

In addition, Average daily gain (ADG) was calculated as the relative amount of daily weight gained by BSF larvae during the experiment, following methods used in animal experiments [11,66], and also frequently reported as a linear growth rate in BSF studies [22,41].

(2) $$\text{ADG}\;\left(\text{mg}/\text{day}\right)=\frac{\text{Final}\;\text{mean}\;\text{larval}\;\text{weight}\;\left(20\;\text{days}\;\text{old}\right)-\text{Initial}\;\text{mean}\;\text{larval}\;\text{weight}\left(6\;\text{days}\;\text{old}\right)}{\text{Experimental}\;\text{duration}\;\left(14\;\text{days}\right)}$$

Larval survival rates (SR %) in different experimental substrates were calculated based on the initial and final larval counts in each rearing container [28].

(3) $$\text{SR}\;\left(\%\right)=\frac{\text{Final}\;\text{larval}\;\text{count}\;\left(20\;\text{days}\;\text{old}\right)}{\text{Initial}\;\text{larval}\;\text{count}\;\left(6\;\text{days}\;\text{old}\right)}\;\times \;100$$

The total weight of the substrate provided during the experiment and the final weight of residue remaining in each container were recorded, both on a fresh and DM basis, to calculate the waste reduction rate (WR), as reported earlier [28].

(4) $$\text{WR}\;\left(\%\right)=\left(1-\frac{\text{Weight}\;\text{of}\;\text{residue}\;\left(\text{day}\;20\right)}{\text{Weight}\;\text{of}\;\text{substrate}\;\text{provided}\;\left(\text{day}\;6\right)}\;\right)\times \;100$$

Both initial and final measurements were also recorded for larval and substrate biomass in each container. The total amount of substrates provided to the BSFL was evaluated for its conversion into larval biomass, residue, and loss in metabolism. Metabolism was determined by subtracting the larval biomass and residue from the total amount of feeding substrate provided as described by Diener et al. [21].

(5) Metabolism=Totalsubstrateprovided−LarvalBiomass−Residueintray

The conversion efficiencies of each substrate by BSFL were evaluated in terms of feed conversion ratio (FCR) and bioconversion rate (BCR) based on the assumption that the BSFL had consumed all provided feeding substrates [49]. BCR and FCR were calculated on a dry matter (DM) basis to ensure consistent comparisons across substrates with varying moisture levels. FCR was estimated as the kilograms of substrates needed to produce a kilogram of insect meal [49].

(6) $$\text{FCR}=\frac{\text{Weight}\;\text{of}\;\text{substrate}\;\text{provided}\;\left(\text{day}\;6\right)}{\text{Final}\;\text{larval}\;\text{biomass}\;\left(\text{day}\;20\right)-\text{Initial}\;\text{larval}\;\text{biomass}\left(\text{day}\;6\right)}$$

BCR, also termed as the efficiency of conversion of ingested food [49], was estimated as the ratio of weight gain to the weight of ingested food [28].

(7) $$\text{BCR}\;\left(\%\;\text{DM}\right)=\frac{\text{Final}\;\text{larval}\;\text{biomass}\;\left(\text{day}\;20\right)-\text{Initial}\;\text{larval}\;\text{biomass}\left(\text{day}\;6\right)}{\text{Weight}\;\text{of}\;\text{substrate}\;\text{provided}\;\left(\text{day}\;6\right)}\;\times \;100$$

### 2.5 Chemical and nutritional analyses of feeding substrates and BSFL

The chemical and nutritional composition of feeding substrates and BSFL were analyzed following AOAC International standards [4]. The DM was determined by drying the samples at 105 °C for 24 hours in a hot air oven. Then, the samples were burned in a muffle furnace (Nabertherm GmbH, Germany) at 550 °C for three hours to determine the ash content (AOAC Method 942.05). For the feeding substrates, the total nitrogen content was determined using the Kjeldahl (COD Digestion, Unilab, India) method (AOAC Method 2001.11) and multiplied with a conversion factor of 6.25 to estimate crude protein. For BSFL samples, the total nitrogen content was determined by the Dumas method (AOAC method 990.03) using an automatic analyzer (Primacas SNC 100-IC-E, Skalar, Netherlands), where a conversion factor of 4.67 was employed to estimate crude protein from total nitrogen, as recommended previously [34]. Crude fat was measured gravimetrically as ether extract using a Soxhlet apparatus (AOAC Method 2003.05). A hot plate instrument (Unilab, India) was used to determine crude fiber content in feeding substrates (AOAC Method 985.25). Whereas for BSFL samples, NDF (Neutral Detergent Fiber) and ADF (Acid Detergent Fiber) were analyzed using the filter bag technique (ANKOM2000 Fiber Analyzer, ANKOM Technology, USA) (AOAC method 2002.4 & 973.18), with BSFL samples being defatted with acetone before the NDF and ADF analyses. Nitrogen-free extract (NFE) in feeding substrates was calculated as 100 – (crude protein + crude fat + ash + crude fiber).

### 2.6 Statistical analyses

Statistical analyses of experimental data were carried out using R Studio (version 2024.04.1) employing general linear model (GLM) techniques. The normality assumption of the GLM method was confirmed with the Shapiro-Wilk test [27]. Treatment combinations of feeding substrates, comprising biowastes with or without rapeseed cake supplementation, were used as independent variables. The effect of feeding substrates on weight gain, survival, bioconversion, and nutritional composition was analyzed using one-way ANOVA, and the effect on larval weight gain was analyzed using ANOVA with repeated measures [58]. Tukey's HSD test was used for multiple comparisons to determine the significant effects of independent variables on treatment means (α < 0.05). Principal Component Analysis (PCA) was performed to analyze the relationships between substrate composition and larval performance. Normalized data on substrate composition and larval performance metrics were subjected to PCA using the prcomp() function in R, with results visualized using a biplot created with the factoextra package. All other graphs and curves were developed using the "ggplot" function from the ggplot2 package. The "growthcurver" and "ggplot2″ packages were used to construct the logistic growth curve of larvae with the "ggplot" function. Parameters for the logistic model (*k,* N_0_, *r*) were estimated using the SummarizeGrowthByPlate function in the R package growthcurver. Predicted values were calculated for smooth logistic growth curves over time.

## 3 Results

### 3.1 Nutritional composition of feeding substrates

The proximate composition of the feeding substrates used to feed BSFL in this study is presented in Table 2. The feeding substrates obtained from fruit and vegetable markets and agricultural farms exhibited generally high moisture levels, exceeding 80 %. CVW collected from fruit and vegetable markets was high in protein (32 % DM) but had very low levels of soluble carbohydrates (NFE: 33 % DM) as compared to BCF (NFE: 65 % DM and protein: 22 % DM). In contrast, BFW and CFW obtained from food processing industries were rich in soluble carbohydrates (NFE: ∼80 % DM) but lower in protein content (<12 % DM) compared to BCF. RDW had the highest fiber content (33 % DM), approximately 3-fold greater than the average fiber content of the other substrates (8 % DM) used in this experiment, followed by BPW with 1.5-fold more fiber (16 % DM) than the average. RSC, a supplement for organic wastes, was rich in both protein (38 %) and fat (8 %) compared to BCF. Overall, food industry wastes (BFW and CFW) were rich in soluble carbohydrates, CVW and RSC were protein-rich wastes, and RDW and BPW were substrates with high fiber content.

**Table 2:** Chemical composition of the organic byproducts used as feeding substrates for black soldier fly larvae (BSFL) in this study.

**Feeding substates**	**Moisture ( %)**	**Crude protein (% DM)**	**Crude fat ( % DM)**	**Crude fiber (% DM)**	**Ash (% DM)**	**NFE (% DM)**
Banana pseudo-stem (BPW)	89.28	2.49	7.29	15.72	22.25	52.25
Mix fruit waste (MFW)	80.81	6.58	5.85	10.28	16.54	60.75
Mix vegetable waste (MVW)	84.68	14.06	2.15	4.05	14.06	65.68
Cowpea-pods waste (CVW)	81.04	32.26	1.50	13.91	18.68	33.65
Bakery waste (BFW)	5.63	8.23	8.18	2.14	3.64	77.81
Chowmein (CFW)	8.58	11.58	1.98	0.82	1.24	84.38
Rapeseed cake (RSC)	5.77	38.05	7.96	11.01	6.57	36.41
Chicken feed (BCF)	10.35	22.44	2.04	3.73	7.09	64.70
Rumen digesta waste (RDW)^1^	78.22	13.5–19.6	0.75	31.9–34.9	11.1–16.2	25.7

1 : [3,48]

### 3.2 Growth performance of BSFL

**Fig. 2:**
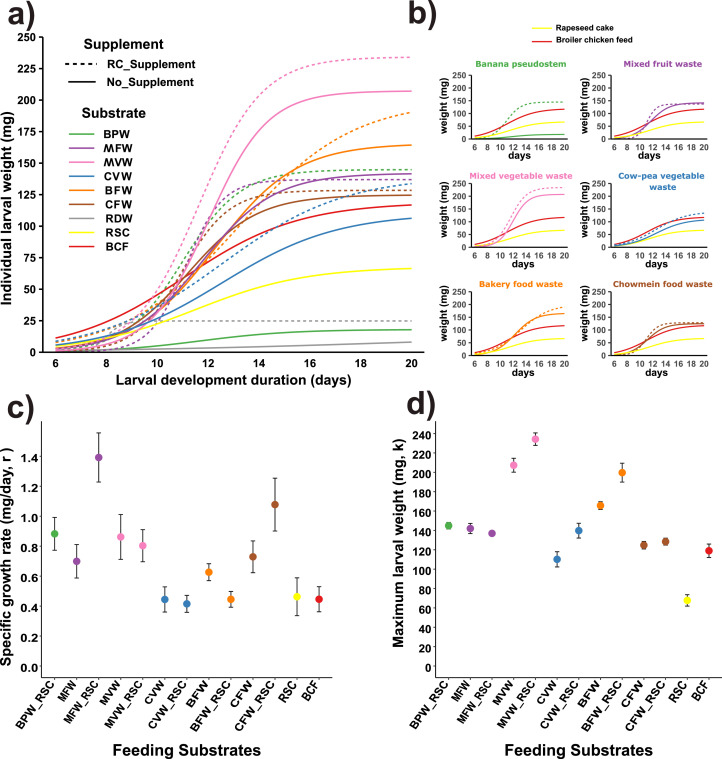
Logistic growth curves **a)** showing BSFL growth trends on all substrates **b)** comparing growth on individual substrates with controls: rapeseed cake (RSC) and broiler chicken feed (BCF) **c)** Specific/logistic growth rate (r) of BSFL **d)** Maximum larval weight (asymptote, K) of BSFL reared on different feeding substrates with and without rapeseed cake supplementation (BPW: banana pseudo-stem waste, MFW: mixed fruit waste, MVW: mixed vegetable waste, CVW: cowpea vegetable waste, BFW: bakery food waste, CFW: chowmein food waste, RDW: rumen digesta waste, RSC: rapeseed cake, BCF: broiler chicken feed,_RSC: biowastes supplemented with 25 % rapeseed cake).

The growth of BSFL was significantly affected by the feeding substrates used in the experiment (p < 0.001). Larvae reared on most biowastes achieved a higher final mean weight than those fed broiler chicken feed (BCF, 133 mg). Similarly, these biowaste-fed larvae exhibited a higher specific growth rate (*r*) than those fed on BCF (0.45 mg/day). In contrast, larvae reared on rapeseed cake (RSC) had a final mean weight of only 65 mg, which was over 50 % lower than that of BCF-fed larvae, despite exhibiting a comparable specific growth rate (*r*) (Fig. 2). However, it was evident from the growth curve (Figs. 2a and b) that BSFL consistently gained more weight throughout the growing period under biowastes supplemented (25 %) with RSC compared to non-supplemented ones, except for BFW. In the case of BFW, RSC supplementation resulted in higher larval weight compared to un-supplemented BFW only after 14 days of larval age. Larvae failed to thrive on rumen digesta waste (RDW) and banana pseudo-stem (BPW) as a minimal weight gain of just about ∼8 mg and ∼16 mg over the entire feeding period of 14 days, leading to a ∼90 % lower final larval weight than that of BCF. While RSC supplementation slightly improved weight gain in larvae reared in BPW, no significant impact of supplementation was observed for those reared on RDW (Fig. 2).

**Fig. 3:**
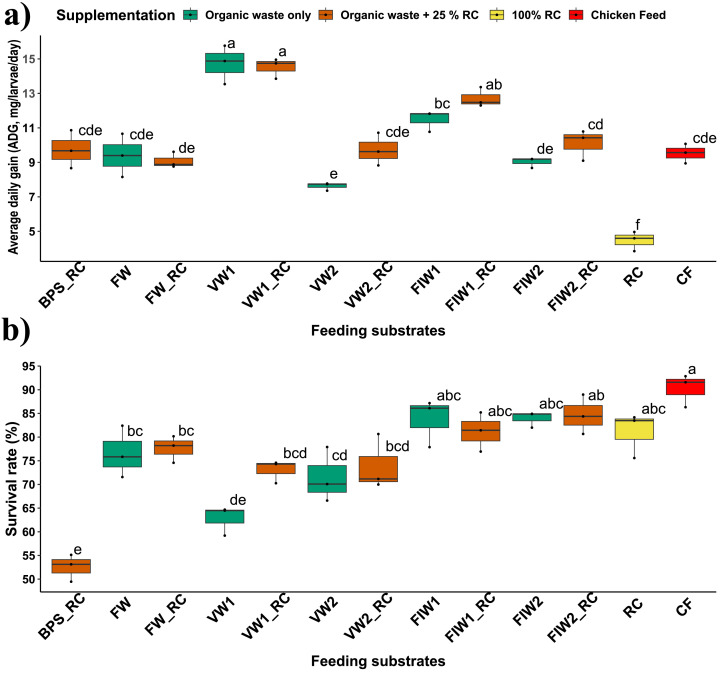
Boxplot showing **a)** average daily weight gain (ADG) and **b)** survival rate of BSF larvae reared on different substrates (BPW: banana pseudo-stem waste, MFW: mixed fruit waste, MVW: mixed vegetable waste, CVW: cowpea vegetable waste, BFW: bakery food waste, CFW: chowmein food waste, RSC: rapeseed cake, BCF: broiler chicken feed, _RSC: biowastes supplemented with 25 % rapeseed cake). Distinct letters indicate significant differences among treatments (p < 0.05), as determined by Tukey's HSD test.

The highest average daily gain (ADG) was observed in larvae reared on MVW, with a final mean weight nearly double that of larvae fed chicken feed (Fig. 2, Fig. 3a). The ADG on MVW was similar to that on MVW+ RSC and BFW+ RSC but significantly higher than on other substrates (p < 0.05 for all) including chicken feed (p < 0.001). In contrast, larvae reared solely on rapeseed cake showed poor growth, with an ADG approximately 50 % lower than those fed chicken feed. However, when rapeseed cake was used as a supplement (25 %) to organic wastes like CVW, BFW, and CFW, a slight improvement (11 to 28 %) in the ADG of BSFL was observed (Fig. 3a). Principle component analysis indicated that the variable that contributed most to the ADG (L_Growth) was the availability of soluble carbohydrates (S_NFE) in substrates (Fig. S1), where a non-linear correlation was obtained between ADG and availability of soluble carbohydrates in feeding substrates (r= 0.47, p= 0.11) (Fig. S2).

### 3.3 Survival of BSFL

The feeding substrates used in this experiment significantly influenced larval survival rates (p < 0.001). BSFL reared on food industry wastes (BFW and CFW) showed higher survival rates (>80 %), which was statistically similar to those fed broiler chicken feed (BCF) (Fig. 3b). In contrast, larvae reared on fruit and vegetable wastes (MFW, MVW, and CVW) had significantly lower survival rates (63–77 %) (p < 0.05 for all) compared to BCF (90 %). Unlike growth performance, BSFL survival rates were not significantly affected when the larvae were reared on 100 % rapeseed cake (RSC) (p= 0.31). Moreover, supplementing MVW with RSC led to a slight increase (16.3 %) in larval survival rates (Fig. 3b).

### 3.4 Waste reduction and bioconversion of BSFL

**Fig. 4:**
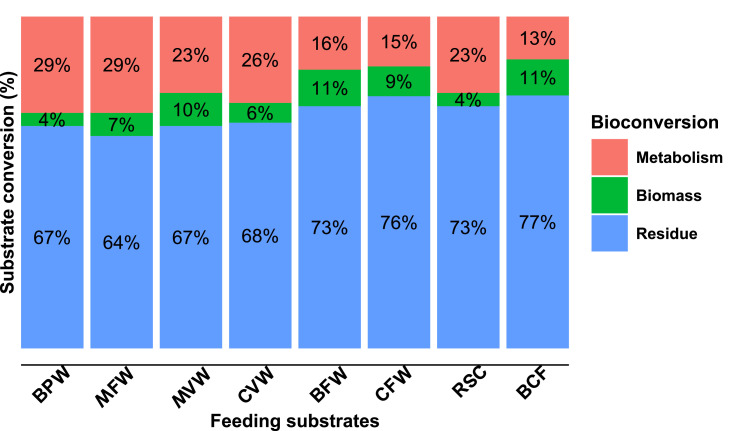
Feeding substrate ( % DM) that is converted to larval biomass, used for metabolism, and then leftover as residues. Metabolism was calculated by subtracting larval biomass and residue from the total amount of feeding substrate provided [21]. BPW: banana pseudo-stem waste, MFW: mixed fruit waste, MVW: mixed vegetable waste, CVW: cowpea vegetable waste, BFW: bakery food waste, CFW: chowmein food waste, RSC: rapeseed cake, BCF: broiler chicken feed.

A significant portion of the feeding substrates (64 % to 77 %) provided to BSFL ended up as residual waste or remained unconsumed by the larvae. Only a tiny fraction (4 % to 11 %) of the organic waste was transformed into larval biomass, while the remaining 13 % to 29 % was lost through larval metabolism (Fig. 4). However, waste reduction rates and conversion efficiencies of organic wastes by BSFL varied significantly across feeding substrates (p < 0.001). BSFL more effectively reduced fruit, vegetable, and agricultural wastes (BPW, MFW, MVW, and CVW) on a fresh weight basis compared to other waste types. However, when assessed on a DM basis, BSFL reared on food industry wastes (BFW and CFW) showed higher waste reduction values, leading to higher bioconversion rates (BCR) (Table 3). The principal component analysis of variables indicated that BCR values were negatively influenced by substrate fiber (S_Fiber; r= -0.84, p < 0.001) and positively influenced by soluble carbohydrates in substrates (S_NFE; r = 0.74, p = 0.003) (Figs S1 and S2). Similarly, the FCR was poorer (> 10) for larvae fed on fruit, vegetable, and agricultural waste compared to the FCR of those fed BCF (9.4). The larvae fed with food industry wastes had FCR comparable to those fed to BCF (Table 3).

**Table 3:** Waste reduction and conversion rates (FCR: Food conversion ratio, BCR: Bioconversion rate), (FW: Fresh weight and DM: Dry matter) of BSF larvae reared on organic wastes with and without rapeseed cake supplementation.

**Feeding substrates**	**Waste reduction (% FW)**	**Waste reduction (% DM)**	**FCR**	**BCR (% DM)**
BPW_RSC	78.9 ± 0.73^a^	67.03 ± 1.7^cde^	22.93 ± 3.13^a^	4.42 ± 0.65^f^
MFW	72.66 ± 1.77^ab^	60.3 ± 4.16^f^	14.55 ± 1.03^b^	6.89 ± 0.49^e^
MFW_RSC	75.95 ± 1.19^ab^	66.75 ± 1.94^cdef^	13.78 ± 1.21^bc^	7.3 ± 0.64d^e^
MVW	78.01 ± 1.06^ab^	65.6 ± 1.48^def^	11.95 ± 1.24^bcd^	8.43 ± 0.9cd^e^
MVW_RSC	78.79 ± 0.76^a^	68.19 ± 0.91^bcde^	9.07 ± 0.52^d^	11.04 ± 0.61^a^
CVW	80.4 ± 2.47^a^	64.85 ± 3.94^def^	20.99 ± 1.85^a^	4.79 ± 0.4^f^
CVW_RSC	83.09 ± 1.04^a^	71.74 ± 0.7^abcd^	14.61 ± 1.36^b^	6.88 ± 0.62^e^
BFW	49.29 ± 5.09d^e^	74.67 ± 2.31^ab^	9.87 ± 0.71^cd^	10.16 ± 0.71^abc^
BFW_RSC	45.44 ± 6.83^e^	71.24 ± 2.91^abcde^	9.16 ± 0.71^d^	10.96 ± 0.82^a^
CFW	59.54 ± 6.93^cd^	75.94 ± 1.55^a^	11.26 ± 0.92^bcd^	8.92 ± 0.7^bcd^
CFW_RSC	47.81 ± 5.38^e^	75.62 ± 1.97^a^	10.15 ± 0.84^cd^	9.9 ± 0.82^abc^
RSC	38.18 ± 5.29^e^	73.1 ± 1.7^abc^	24.08 ± 2.06^a^	4.17 ± 0.34^f^
BCF	67.15 ± 1.16^bc^	76.66 ± 0.86^a^	9.42 ± 0.54^d^	10.64 ± 0.62^ab^

Data are presented as mean ± SD, n = 3. Distinct letters within the same columns indicate significant differences among treatments (p < 0.05), as determined by Tukey's HSD. BPW: banana pseudo-stem waste, MFW: mixed fruit waste, MVW: mixed vegetable waste, CVW: cowpea vegetable waste, BFW: bakery food waste, CFW: chowmein food waste, RSC: rapeseed cake, BCF: broiler chicken feed, _RSC: biowastes supplemented with 25 % rapeseed cake.

In general, no significant difference was observed in WR between BSFL reared on biowastes with or without RSC supplementation. However, the BCR of BSFL reared on vegetable-based wastes (MVW and CVW) and the FCR of larvae reared on CVW improved significantly (p < 0.05 for all) with RSC supplementation compared to those without supplementation (Table 3). Conversely, larvae fed exclusively on RSC as a substrate had very poor conversion efficiencies (FCR: 24.1, BCR: 4.1) compared to those fed on BCF (FCR: 9.4, BCR: 10.6).

### 3.5 Nutritional composition of BSFL

Rearing substrates significantly affected the protein, fat, ash, and fiber (NDF and ADF) content of BSF larvae (p < 0.001 for all). The moisture contents of larvae were higher in larvae (77–78 %) reared in the feeding substrates with higher moisture, such as agriculture, fruit, and vegetable wastes (BPW, MFW, MVW, and CVW) compared to those (69–73 %) reared on low moisture containing substrates (BFW, CFW, RSC, and BCF) (TableS. 2 and 4).

**Table 4:** Moisture, Ash, Neutral Detergent Fiber (NDF), and Acid Detergent Fiber (ADF) content of BSF Larvae reared on different organic waste as feeding substrates.

**Feeding substrates**	**Moisture (%)**	**Ash (% DM)**	**NDF (% DM)**	**ADF (% DM)**
BPW_RSC	78.02 ± 0.71^a^	18.13 ± 1.53^b^	18.93 ± 0.65^ab^	13.78 ± 1.19^abc^
MFW	75.77 ± 1.65^abc^	14.6 ± 1.38^c^	14.44 ± 1.28^cd^	10.47 ± 0.84^cde^
MFW_RSC	74.06 ± 0.1^abcd^	13.79 ± 1.34^cd^	16.87 ± 0.82^bc^	11.08 ± 0.91^cd^
MVW	77.03 ± 1.81^ab^	19.77 ± 0.98^ab^	15.24 ± 1.66^bc^	11.38 ± 1.69^bcd^
MVW_RSC	73.85 ± 1.64^bcde^	18.42 ± 0.62^b^	15.31 ± 1.45^bc^	11.11 ± 0.92^cd^
CVW	77.77 ± 0.94^ab^	22.49 ± 0.85^a^	22.78 ± 1.24^a^	15.86 ± 1.2^a^
CVW_RSC	75.84 ± 0.77^ab^	20.09 ± 0.69^ab^	22.66 ± 2.14^a^	14.57 ± 1.29^ab^
BFW	73.47 ± 0.53^bcde^	11.4 ± 1.28^de^	10.28 ± 1.7^de^	5.65 ± 1.49^g^
BFW_RSC	73.42 ± 0.81^bcde^	10.64 ± 0.65^e^	12.97 ± 1.4^cde^	8.15 ± 1.08^defg^
CFW	70.57 ± 0.72^def^	6.2 ± 0.56^f^	10.17 ± 1.49^de^	7.07 ± 1.44^efg^
CFW_RSC	71.01 ± 1.95^cdef^	5.45 ± 0.09^f^	9.77 ± 1.18^e^	6.54 ± 0.84^fg^
RSC	71.03 ± 0.13^ef^	11.1 ± 0.28^de^	13.59 ± 1.61^cde^	9.24 ± 1.17^def^
BCF	69.09 ± 0.95^f^	10.46 ± 0.09^e^	15.16 ± 1.55^bc^	8.92 ± 0.52^defg^

Data are presented as mean ± SD, n= 3. Distinct letters within the same columns indicate significant differences among treatments (p < 0.05), as determined by Tukey's HSD. BPW: banana pseudo-stem waste, MFW: mixed fruit waste, MVW: mixed vegetable waste, CVW: cowpea vegetable waste, BFW: bakery food waste, CFW: chowmein food waste, RSC: rapeseed cake, BCF: broiler chicken feed, _RSC: biowastes supplemented with 25 % rapeseed cake. DM: dry matter

**Fig. 5:**
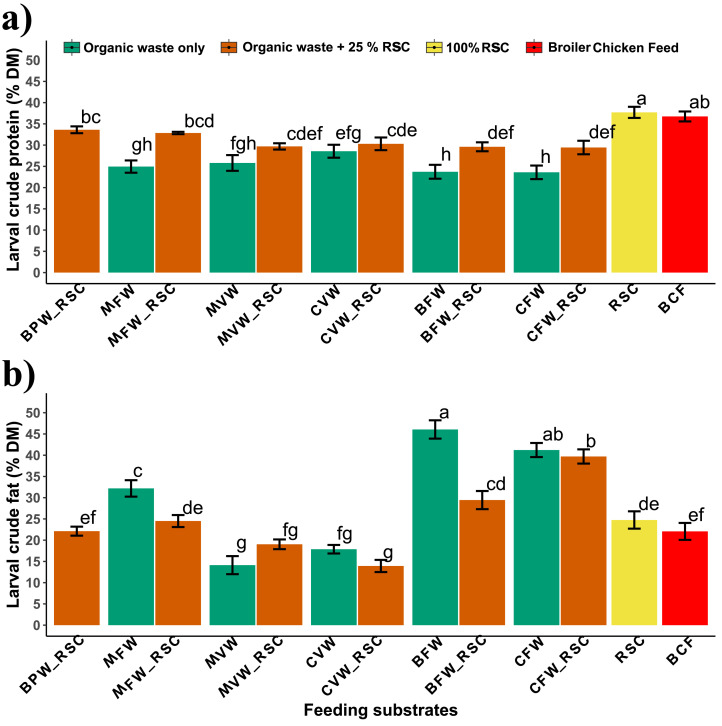
a) Crude protein **b)** Crude fat (Mean ± SD, *n*= 3) content of BSF larvae reared in different organic wastes as affected by feeding substrates and rapeseed cake supplementation (BPW: banana pseudo-stem waste, MFW: mixed fruit waste, MVW: mixed vegetable waste, CVW: cowpea vegetable waste, BFW: bakery food waste, CFW: chowmein food waste, RSC: rapeseed cake, BCF: broiler chicken feed, _RSC: biowastes supplemented with 25 % rapeseed cake.). Distinct letters indicate significant differences among treatments (p < 0.05), as determined by Tukey's HSD test.

The highest crude protein content (37.7 % DM) was found in larvae reared on 100 % rapeseed cake (RSC) and was statistically similar to those fed BCF (36.7 % DM) (p= 0.99) (Fig. 5a). Besides RSC and BCF, the next highest crude protein content (28.6 % DM) was found in larvae reared on CVW, but this was significantly lower (p < 0.001) than that of larvae reared on BCF (p < 0.001) and RSC (p < 0.001). Regarding fat content, the larvae reared on industrial food wastes (BFW and CFW) had the highest crude fat contents (46.1 % and 41 % DM, respectively), both being significantly higher than those reared on control substrates (BCF and RSC) or other organic wastes (p < 0.001 for all) (Fig. 5b). Overall, larvae reared on food industry-based waste had lower crude protein content (∼24 % DM), while those fed vegetable wastes (MVW and CVW) had lower crude fat content (14–19 % DM). Ash and fiber contents were highest in larvae reared on vegetable waste (CVW), followed by other agriculture, fruit, and vegetable waste (Table 4). A strong correlation was observed between the ash (r = 0.89, p < 0.001) and protein (r = 0.57, p = 0.04) content in larvae and their feeding substrates. Similarly, the fat content in larvae was closely associated with the soluble carbohydrates in the substrates (r = 0.72, p= 0.005) (Figs S1 and S2).

Supplementing organic wastes with rapeseed cake significantly affected the protein and fat content of BSF larvae (Fig. 5). The protein content of larvae increased significantly with the supplementation (25 %) of rapeseed cake to biowastes such as MFW (p < 0.001), BFW (p < 0.001), and CFW (p < 0.001), as compared to the larva reared on their respective un-supplemented wastes. Conversely, the fat content in larvae decreased significantly when MFW (p < 0.001) and BFW (p < 0.001) were supplemented with rapeseed cake compared to non-supplemented ones. A similar trend of slightly higher protein and lower fat content due to supplementation was observed across other wastes, except in the case of MVW, where fat content increased slightly (p = 0.07) with supplementation. Rapeseed cake supplementation did not significantly affect the larvae's ash, fiber, and moisture content. Nonetheless, a similar trend of numerical decrease in ash content was observed across all organic wastes with supplementation (Table 4).

## 4 Discussion

Efficient and environmentally sustainable recycling technologies are urgently needed in Global South countries to address the serious challenges of organic solid waste management. To the best of our knowledge, this study, for the first time aimed to evaluate: a) the suitability of recycling commonly produced organic waste fractions in Nepal using BSFL, and b) the potential of rapeseed cake supplementation to enhance larval growth performance and nutritional composition. The findings from this study evidently demonstrated that most organic wastes available in urban regions of Nepal, except rumen digesta waste and banana pseudo-stem, were suitable for bioconversion using BSFL. However, larval growth, survival, nutritional values, and bioconversion efficiency varied widely depending on the types of organic waste used to feed them. Additionally, supplementing organic wastes with rapeseed cake was found to be an effective dietary strategy to optimize larval performance and nutritional composition.

### 4.1 Biowastes differentially influence the growth, survival, and bioconversion efficiency of BSFL

The growth and development of BSFL greatly depend on the feeding substrates provided [45]. In this study, BSFL performed remarkably well in terms of their growth and bioconversion efficiency when fed with biowastes from food industries and fruit and vegetable markets with similar larval weight gain compared to those fed chicken feed. Results from this study indicated that variations in the growth and bioconversion efficiency of BSFL were primarily influenced by the presence of soluble carbohydrates in the feeding substrates. For example, biowastes rich in digestible carbohydrates like chowmein, bakery, and mixed fruit waste led to higher larval growth, which could likely be due to the conversion of excess carbohydrates into fat via *de novo* lipogenesis as evidenced by higher fat contents in larvae fed these substrates. In agreement with our findings, substrates rich in digestible carbohydrates have been shown to enhance the growth rate and final larval weight in previous studies [8,15]. Additionally, the greater larval weight gain was linked to higher bioconversion efficiency, particularly in those larvae fed food industry wastes rich in soluble carbohydrates, such as bakery and chowmein wastes. These findings indicate most organic wastes generated from food industries and fruit/vegetable markets can supply balanced nutrients to BSFL, making them just as effective as commercial chicken feed for BSFL production. Overall, the study demonstrated that biowastes from the food industries and fruit and vegetable markets could be ideal feeding substrates for optimal BSFL growth and biomass production.

Findings from this study highlighted that feeding substrates rich in fiber content can negatively affect both the growth and bioconversion efficiency of larvae. For instance, BSFL struggled to thrive on rumen digesta waste and banana pseudo-stem waste due to a high level of complex non-digestible fiber fractions like lignin and cellulose in these substrates [16,57]. These complex fibers make it difficult for BSFL to digest and extract essential nutrients from substrates [19,67]. As these two biowastes are substantially available in Nepal and other South-Asian regions, their utilization by BSFL could perhaps be improved by certain pre-treatments prior to feeding to larvae. Interestingly, it has shown that the utilization of palm oil industry side streams by BSFL could be improved by partial breakdown of fibers via microbial (fungal) fermentation, such as using smoky bracket fungus (*Bjerkandera adusta*), as it promoted the digestibility of the substrates and growth and development of BSFL [37]. Moreover, nitrogen supplementation has been shown to accelerate the bioconversion of high-fiber organic wastes by decreasing the carbon-to-nitrogen ratio and promoting gut microorganisms that aid in fiber digestion [50]. In this study, along with BSW and RDW, other fruit and vegetable wastes also had a relatively high fiber level, leading to low larval bioconversion efficiency. Taken together, while organic wastes rich in soluble carbohydrates support optimal growth and development of BSFL, substrates high in structural carbohydrates, such as lignin and cellulose, can diminish the growth performance of larvae, resulting in lower larval biomass accumulation. However, the application of suitable chemical or microbial pre-treatments can enhance larval growth and improve the bioconversion efficiency of larvae under fiber-rich biowaste and byproducts.

The growth of BSFL largely depends on the availability of nutrients in balanced proportions, particularly the protein:carbohydrate (P:C) ratio in feeding substrates [7,15,28]. For example, Barragan‐Fonseca et al. [8] studied combinations of protein and carbohydrate in BSFL diets and found that carbohydrates had a more significant influence on larval weight than protein and recommended a P:C ratio of 1:3 for optimal performance. Similarly, this study also found that rapeseed cake and cowpea vegetable waste produced lower larval weight than chicken feed despite their high protein contents, likely due to a lower level of digestible carbohydrates in those substrates. Eggink et al. [25] also observed slower growth of BSFL reared on low-carbohydrate, protein-rich substrates like shrimp waste, mitigation mussels, and rapeseed cake. They further suggested that larvae relied on protein as their primary energy source, which is less efficient than utilizing carbohydrates. Additionally, the slower growth of larvae on rapeseed cake could be due to a highly viscous texture of the substrates, as a compact layer was observed at the top layer of the substrate in this study, which could restrict larval movement and feed utilization. Similar observations were also reported by [32], as they found that full oil cake diets extended the larval duration and reduced their prepupal weight, likely due to the compactness of the substrate.

In the present study, supplementing food wastes containing high carbohydrates and low-protein levels, such as bakery and chowmein wastes, with protein-rich rapeseed cake improved BSFL growth, potentially due to a balanced supply of protein and soluble carbohydrates. Chia et al. [18] also reported higher BSFL and prepupal weight when reared on agro-industrial byproducts supplemented with a protein-rich diet. Such improved larval growth due to supplementation could be due to a better P:C ratio of 1:2 or 1:3 in the substrates, as previously suggested [8,24]. These findings indicate that substrates rich in protein but low in carbohydrates, such as rapeseed cake and cowpea vegetable waste, are not favorable for BSFL growth alone. However, such protein-rich bioresources can be effectively utilized to supplement biowastes that are high in soluble carbohydrates but low in protein contents, improving the P:C ratio of the substrates and subsequently promoting BSFL growth and biomass production.

The moisture content of substrates can have a greater impact on BSFL performance and survival than substrate composition [15,60]. In this study, BSFL reared on food industry biowastes adjusted to a moisture content of 70 % had survival rates comparable to larvae fed chicken feed. In contrast, larvae reared on high-moisture biowastes (>80 %), such as fruit and vegetable waste, had poor survival rates. Lalander et al. [40] also reported a reduced survival rate as substrate moisture content increased from 76 % to 97.5 %, and Bekker et al. [9] observed larval death at more than 85 % substrate moisture. Visual observations of larvae trying to avoid the high-moisture containing substrates during the experiment also suggested their discomfort with excessive moisture levels. Although the high moisture content of fruit and vegetable wastes appears to pose challenges for their use as a BSFL-rearing substrate, these biowastes can be effectively utilized by adding dry, fiber-rich materials like rice husks, as suggested by Laksanawimol et al. [39]. Such low-cost agricultural byproducts can absorb excess moisture from the substrate, making it more suitable to get recycled by BSFL. In addition, the fiber present in it can improve the substrate's physical properties by increasing water retention and enhancing porosity, creating optimal conditions for larval development [9,69]. In conclusion, substrates with high moisture content, such as fruit and vegetable wastes, are less favorable for BSFL survival, however, their suitability can be effectively improved by adding dry, fiber-rich materials to balance the moisture level and create a favorable condition for larval development and survival.

### 4.2 The chemical composition of biowastes influences the nutritional values of BSFL

The nutritional composition of BSFL is primarily influenced by the composition of the substrates used to rear them [33,53]. This study found a similar trend as the ash and protein contents of larvae were closely related to the levels present in the feeding substrates, while fat content was associated with the content of soluble carbohydrates in the substrates. Specifically, BSFL reared on high-protein substrates, such as rapeseed cake, had higher protein levels, and those reared on mineral-rich substrates, like fruit and vegetable waste, contained higher mineral levels, and feeding food industry waste rich in soluble carbohydrates resulted in higher larval fat content. The PCA and correlation analysis further illustrated that fat and mineral contents in BSFL can be modified to a large extent through dietary modulation. These findings are consistent with a previous study, which demonstrated that substrate composition had a greater influence on larval fat and ash contents than protein, and carbohydrate-rich substrates led to a higher fat content in larvae (Spranghers et al. [62].

BSFL can store carbohydrates in the form of glycogen, but they can *de novo* synthesize fat from excess carbohydrates and store it as reserve energy in the form of triglycerides within adipocytes, which are capable of holding large amounts of fats as cytoplasmic lipid droplets [5]. Interestingly, an inverse relationship was observed in this study between fat and protein content in the larvae, where higher fat content corresponded with lower protein content and vice versa. This pattern has been observed in other studies as well [17,43,45], which may be due to metabolic trade-offs during nutrient assimilation and storage in the larval body. For example, low-protein diets such as food industry wastes in this study tended to promote fat accumulation, leading to lower protein content, while high-protein diets such as rapeseed cake could support protein accumulation at the expense of fat storage [52]. This is because BSFL can adjust their metabolism in response to changes in dietary compositions, which is regulated by differential expression of key genes, such as hexamerin genes, involved protein and lipid accumulation and mobilization [12,52]. This study also found that supplementing low-protein biowastes, such as food industry and mixed fruit/vegetable wastes, with a protein-rich substrate like rapeseed cake slightly increased the protein content and decreased the fat content in the larvae, suggesting a metabolic shift towards a high protein accumulation at the expense of fat deposition [17]. Taken together, this study indicates the importance of substrate composition in optimizing the nutritional values of BSFL for future food/feed applications.

In conclusion, substrates rich in carbohydrates, such as food industry waste, promote fat accumulation, while protein-rich diets, such as rapeseed cake, support higher protein deposition in the larval biomass. However, high-protein substrates like rapeseed cake do not seem to be suitable as a sole-rearing substrate for BSFL, likely due to their low soluble carbohydrate content. Instead, protein-rich byproducts and bioresources can effectively be utilized to supplement carbohydrate-rich biowastes, enhancing both larval growth and nutritional composition. Overall, these findings imply that shaping a favorable P:C ratio while optimizing the moisture content of substrates is critical for improving the growth performance, survival, and nutritional composition of BSFL. Future studies are needed to clarify any potential risk of chemical or microbial safety associated with the further application of BSFL raised upon locally available diverse bioresources, including biowastes and byproducts. Nevertheless, this study further highlights that BSFL can serve as a versatile and effective biological tool to mitigate alarming solid biowaste management challenges in the developing world while positively contributing to future feed security.

## 5 Conclusions

This study evaluated the suitability of various organic wastes commonly produced in Nepal for both waste recycling and BSFL biomass production in light of poor self-sufficiency in domestic protein supply and critical challenges in biowaste handling in the country. The findings demonstrate that most organic wastes found in Nepal, particularly those from food industries and fruit/vegetable markets, are suitable for BSFL production. Biowastes rich in soluble carbohydrates, such as bakery waste, chowmein waste, and mixed vegetable waste, supported higher larval growth and bioconversion efficiency while promoting larval fat deposition. High-protein substrates, such as rapeseed cake, did not support BSFL growth as a sole rearing substrate, but their supplementation could improve the P:C ratio of carbohydrate-rich substrates, enhancing the growth performance and nutritional quality (protein content) of larvae. On the other hand, this study indicates that high-fiber containing biowastes, such as banana pseudo-stem and rumen digesta, probably require suitable pre-treatments before exposing them to BSFL to enhance digestibility and nutrient availability. Additionally, the moisture content of biowastes obtained from agriculture farms and fruit/vegetable markets needs to be carefully adjusted to improve BSFL survival and development. Overall, a careful selection or combination of available feeding resources is required to achieve optimal chemical composition and a favorable P:C ratio of substrates for BSFL production. This study clearly demonstrates the potential use of BSFL as an effective biological tool to mitigate serious challenges of solid biowaste management in Nepal and other developing countries while positively contributing to future feed security. Future studies are needed to clarify any potential risk of chemical or microbial safety associated with the further application of BSFL.

## Glossary

**ADG:** Average Daily Gain. Average weight gained by larvae throughout the experimental period.

**BSF:** Black Soldier Fly (*Hermetia illucens*), an insect species used for the bioconversion of organic wastes into high-quality protein and fat-rich biomass.

**BSFL:** Black Soldier Fly Larvae, the larval stage of BSF,

**BCR:** Bioconversion Rate, the efficiency of converting feed into larval biomass, expressed as a percentage of dry matter.

**BCF:** Broiler Chicken Feed, commercial poultry feed used as a control substrate in BSFL feeding experiments.

**BPW:** Banana Pseudo-stem Waste, a fibrous agricultural biowaste collected from banana orchards.

**BFW:** Bakery Food Waste, a carbohydrate-rich substrate derived bread cuttings and expired breads collected from local bakery.

**CFW:** Chowmein Food Waste, a carbohydrate-rich food industry byproduct derived from expired chowmein.

**CVW**: Cowpea Vegetable Waste, a protein-rich substrate derived from wasted green cowpea pods collected from vegetable markets.

**DM:** Dry Matter, the portion of a substrate or larval biomass excluding moisture content.

**FCR:** Feed Conversion Ratio, the amount of feed required to produce one unit of larval biomass.

**L_...:** Prefix used in figures for variables related to larval performance (e.g., L_Growth for larval weight gain).

**MFW:** Mixed Fruit Waste, a carbohydrate-rich substrate derived from waste with 50 % banana and 50 % watermelon sourced from fruit markets.

**MVW:** Mixed Vegetable Waste, a carbohydrate and protein-rich substrate derived from waste with 50 % potato, 25 % brinjal, and 25 % tomato collected from vegetable markets.

**NFE:** Nitrogen-Free Extract, a measure of soluble carbohydrates in feed substrates contributing to energy storage.

**P:C Ratio:** Protein-to-Carbohydrate Ratio, critical for optimal growth and nutritional quality of BSFL.

**RSC:** Rapeseed Cake, a byproduct of rapeseed oil extraction, used as a protein supplement

**RDW:** Rumen Digesta Waste, a fibrous byproduct inside the rumen of buffalo discarded while slaughtering, collected from a local slaughterhouse.

**WR:** Waste Reduction Rate, the percentage reduction of waste biomass through BSFL processing.

## Funding

This study was supported by the CEER project (Project number: 2021/10,345)” funded by the Norwegian Agency for International Cooperation and Quality Enhancement in Higher Education (HK-dir.) under the Norwegian Partnership Program for Global Academic Cooperation (NORPART).

## CRediT authorship contribution statement

**Bhola Gautam:** Writing – review & editing, Writing – original draft, Visualization, Software, Methodology, Investigation, Formal analysis, Data curation, Conceptualization. **Sundar Tiwari:** Writing – review & editing, Supervision, Project administration, Methodology. **Min Raj Pokhrel:** Writing – review & editing, Supervision, Methodology. **Jeffery K. Tomberlin:** Writing – review & editing, Validation, Data curation. **Prabhat Khanal:** Writing – review & editing, Supervision, Resources, Project administration, Methodology, Funding acquisition, Conceptualization.

## Declaration of competing interest

The authors declare that they have no known competing financial interests or personal relationships that could have appeared to influence the work reported in this paper.

## Data Availability

Data will be made available on request.

## Appendix Supplementary materials

Image, application 1

Image, application 2

Image, application 3
