# Supplementary figures and images for: Expanding black soldier fly (BSF; Hermetia illucens; Diptera: Stratiomyidae) in the developing world: Use of BSF larvae as a biological tool to recycle various organic biowastes for alternative protein production in Nepal
Source: Biotechnol Rep (Amst). 2025 Feb 10;45:e00879. doi: 10.1016/j.btre.2025.e00879 (PMC11879680; doi:10.1016/j.btre.2025.e00879)

PCA Biplot of Substrate Impact on BSF Larvae

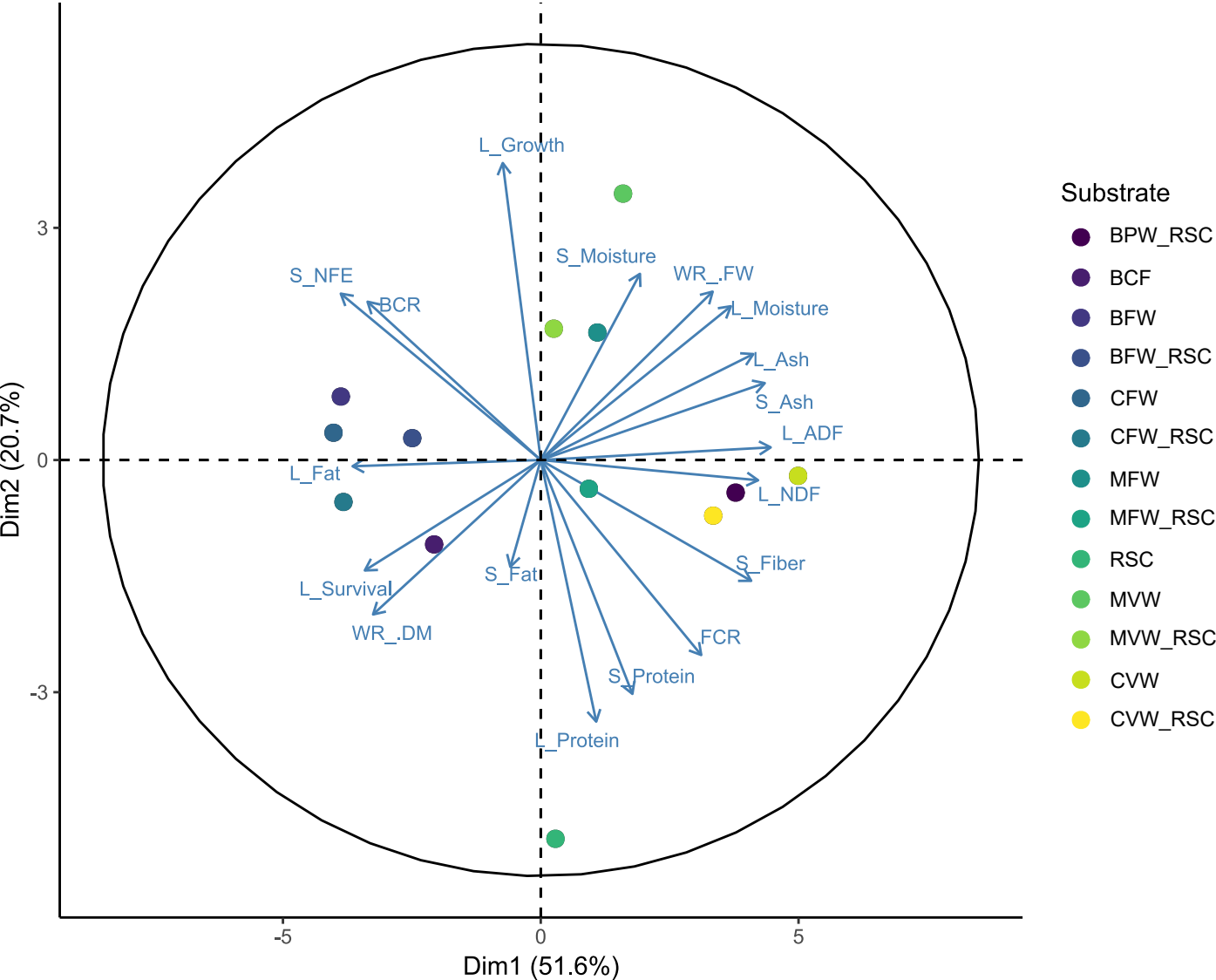

Supplement: Supplementary file 1 [file mmc1.pdf]

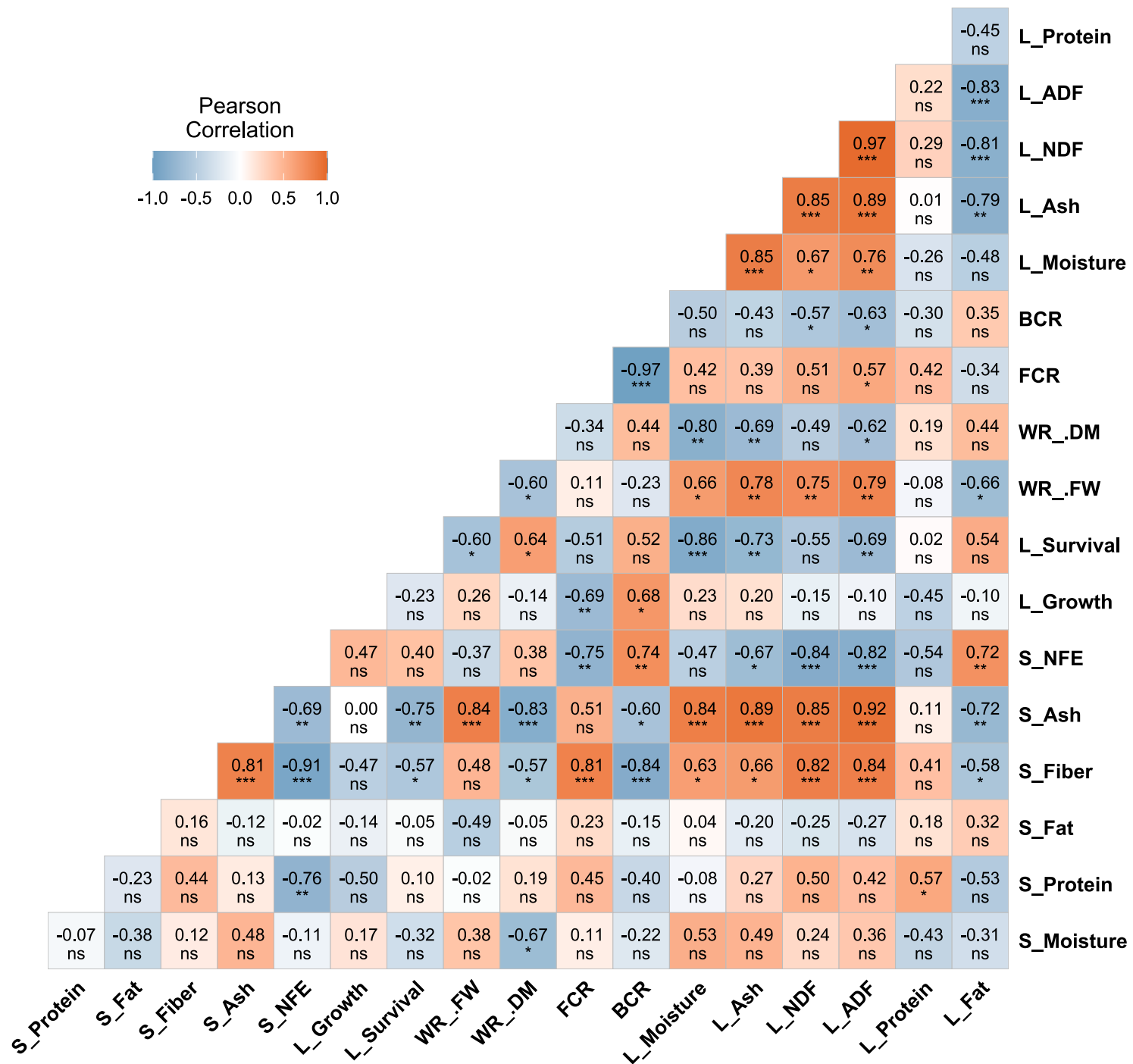

ns  $p \geq 0.05$ ; \*  $p < 0.05$ ; \*\*  $p < 0.01$ ; and \*\*\*  $p < 0.001$

Supplement: Supplementary file 2 [file mmc2.pdf]
