# Supplementary material for: Expanding black soldier fly (BSF; Hermetia illucens; Diptera: Stratiomyidae) in the developing world: Use of BSF larvae as a biological tool to recycle various organic biowastes for alternative protein production in Nepal
Source: Biotechnol Rep (Amst). 2025 Feb 10;45:e00879. doi: 10.1016/j.btre.2025.e00879 (PMC11879680; doi:10.1016/j.btre.2025.e00879)
Supplement: Supplementary file 3 [file mmc3.docx]

**Figure S1.** PCA Biplot representing the impact of feeding substrates composition (S_...) on larval performance (L_....) when reared in different feeding substrates (BPW: banana pseudo-stem waste, MFW: mixed fruit waste, MVW: mixed vegetable waste, CVW: cowpea vegetable waste, BFW: bakery food waste, CFW: chowmein food waste, RSC: rapeseed cake, BCF: broiler chicken feed, _RSC: biowastes supplemented with 25% rapeseed cake). DM: dry Matter, FW: fresh weight.

**Figure S2.** Correlation-plot sowing relationship between composition of feeding substrates (S_...) and larval (L_....) growth, survival, bioconversion, and composition.
